# Long-term outcomes following lateral alveolar ridge augmentation using a collagenated xenogeneic bone block: a monocenter, prospective single-arm clinical study

**DOI:** 10.1186/s40729-021-00293-3

**Published:** 2021-02-22

**Authors:** Frank Schwarz, Didem Sahin, Sara Civale-Schweighöfer, Jürgen Becker

**Affiliations:** 1 Department of Oral Surgery and Implantology, Goethe University, Carolinum, Frankfurt, Germany; 2 Department of Oral Surgery, Universitätsklinikum Düsseldorf, Düsseldorf, Germany

**Keywords:** Clinical study, Alveolar ridge augmentation, Collagenated xenogeneic bone block, Histological analysis

## Abstract

**Objectives:**

To assess the long-term clinical outcomes following lateral alveolar ridge augmentation using a collagenated xenogeneic bone block (CXBB) and staged implant placement.

**Material and methods:**

A total of *n* = 9 patients (9 implants) were available for the analysis. Each subject had received lateral ridge augmentation using a size-adapted rigidly fixed CXBB and contour augmentation at single-tooth gaps. Implant placement was performed after 24 weeks of submerged healing. Clinical parameters (e.g., bleeding on probing (BOP), probing pocket depth (PD), mucosal recession (MR)) were recorded at 16 to 20 weeks after the cementation of the crown (baseline) and scheduled for 0.5 (visit 1 (V1)), 1.5 (V2), 2.5 (V3), 3.5 (V4), and 4.5 (V5) years after implant loading.

**Results:**

Changes in clinical parameters commonly remained low throughout the entire observation period. Significant changes to baseline were merely noted for mean BOP scores at V4 (19.14 ± 17.75%; *n* = 7; *P* = 0.029) and mean PD scores at V2 (0.78 ± 0.98 mm; *n* = 9; *P* = 0.044) and V3 (1.33 ± 1.05 mm; *n* = 9; *P* = 0.009), respectively.

**Conclusion:**

CXBB was associated with high clinical implant success and survival rates on the long-term.

## Introduction

Staged lateral bone augmentation is a commonly used clinical procedure to allow for implant placement in an ideal prosthetic position to ensure function and aesthetics [1]. Different materials including autogenous bone, xenografts, allografts, alloplasts, or combination of these have been investigated and proven to be associated with an overall weighted mean clinical bone width gain of 3.45 ± 1.18 mm [2]. However, the incidence of complications was high, with wound dehiscences and premature membrane/graft exposures being among the most frequent events (i.e., 5–54%). This was less frequently (i.e., 7–13%) associated with wound infections and a loss of the graft material. In addition, harvesting of autogenous bone grafts was frequently (9–66%) associated with a transient paresthesia [2]. Accordingly, it was recommended that patient morbidity should play a crucial component when selecting treatment options [1].

Previously, a screwable, collgenated xenogeneic bone block (CXBB) of equine origin was introduced as an alternative scaffold for lateral bone augmentation prior to implant placement. Preclinical animal studies provide evidence that CXBB offers promising osteoconductive properties to promote bone ingrowth and a timely graft remodeling in various defect models [3–5]. The safety and efficacy of CXBB for staged lateral grafting has also been confirmed in clinical studies. At 24 weeks, the reported mean gain in ridge width ranged between 3.88 ± 1.75 mm and 4.12 ± 1.18 mm, thus enabling implant placement at the majority of the sites investigated [6, 7]. Limited clinical data also pointed to stable and healthy peri-implant soft tissue conditions after a short-term loading period of 16–20 weeks [6].

The aim of this prospective single arm clinical follow-up study was to assess the long-term clinical outcomes following lateral alveolar ridge augmentation using CXBB and staged implant placement.

## Material and methods

### Study design and participants

This study reports on the 4.5-year clinical outcomes of a monocenter, prospective single-arm clinical study, which aimed at evaluating the safety and efficacy of CXBB for lateral alveolar ridge augmentation and two-stage implant placement.

A total of 9 patients (5 women, 4 men; mean age 49.4 years; range between 36 and 72 years; mean weight 72.4 kg; range between 52 and 110 kg) were included.

The study protocol including the extended observation period was approved by the ethics committee of the Heinrich Heine University, Düsseldorf, Germany.

The clinical investigation including monitoring was performed according to ISO GCP 14155:2011. The present reporting considered the checklist items as proposed in the STROBE statement.

### Inclusion and exclusion criteria

The Subjects were included in the study if they present all of the following conditions: (1) successful lateral augmentation in the initial study [6], (2) successful implant placement at the augmented site, (3) final restoration, and (4) written informed consent.

The subjects were not included in the study if they present one of the following conditions: (1) occurrence of newly diagnosed diseases interfering with hard tissue regeneration; (2) history of a trauma to the implant site; (3) history of orthodontic treatment in the same quadrant; (4) immunosuppressant, corticosteroid or bisphosphonate therapy; (5) inflammatory and autoimmune disease of the oral cavity; (6) history of malignancy, radiotherapy, or chemotherapy for malignancy within the past 5 years; (7) smokers (> 10 cigarettes per day); (8) insulin dependent diabetes; (9) pregnant or lactating women; or (10) participation in a clinical study interfering with the objective of this follow-up observation.

### Sample size calculation

Due to the proof of principle character of the initial pilot study and lack of reference data in the literature, a sample size calculation was neither reasonable nor feasible. A sample size of *n* = 10 was considered to be sufficient to allow a first evaluation of the safety and performance of CXBB in exploratory study [6]. Nine patients were eligible to enter the long-term follow-up observation.

### Treatment procedures

**Fig. 1:**
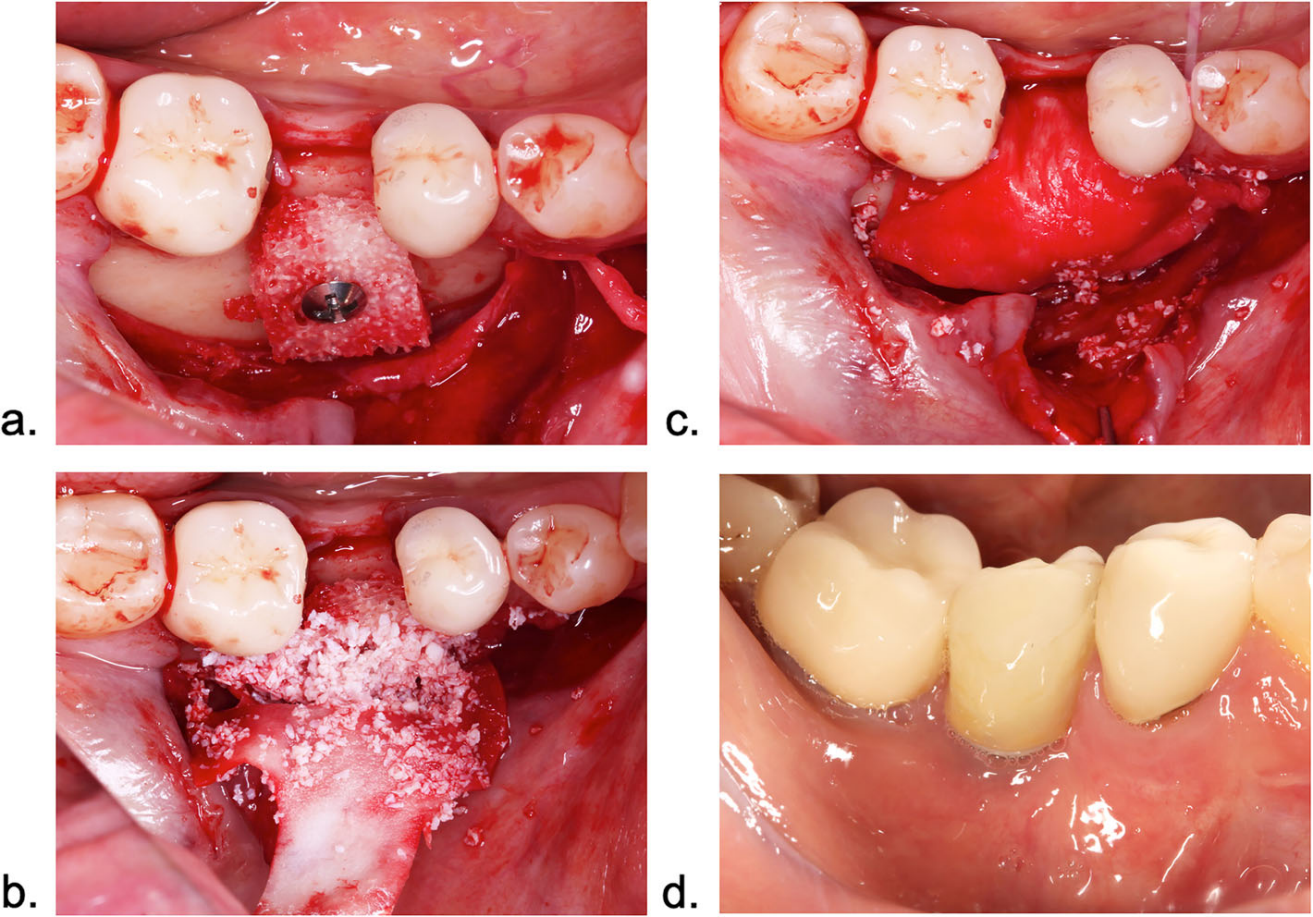
Surgical procedure and follow-up visits. **a** Lateral ridge augmentation using CXBB. A central osteosynthesis screw was used for graft fixation. **b** Contour augmentation using DBBM + NBCM. **c** Flap advancement to account for a submerged healing procedure. **d** Clinical baseline measurements were recorded at 16 to 20 weeks after the cementation of the crown and at V1–5

The surgical procedures have been reported in detail previously [6]. In brief, mucoperiosteal flaps extending to and including the adjacent teeth were prepared to expose the respective target sites. One rehydrated (i.e., sterile saline solution for 30 s) single CXBB (dimensions 10 × 10 × 5 mm) (Geistlich Bio-Graft®, Geistlich Pharma AG, Wolhusen, Switzerland) was used per site. Prior to fixation using one titanium osteosynthesis screw (1.5 × 9 mm, Medicon, Tuttlingen, Germany), the predrilled blocks were shaped to match the size and shape of the defect area. The peripheral part of CXBB was covered by a deproteinized bovine bone mineral (Geistlich Bio-Oss®, Geistlich Biomaterials, small granules) (DBBM) along with the application of a native bilayer collagen membrane applied in a double layer (Geistlich Bio-Gide®, Geistlich Biomaterials) (NBCM). Coronally advanced flaps were repositioned to ensure a tension-free submerged healing (Fig. 1a–c).

At 24 weeks, commercially available titanium implants (Bone Level® SLActive®, Institut Straumann AG, Basel, Switzerland) were inserted in an epicrestal position. Due to an insufficient gain in ridge width, two patients had do undergo an additional (i.e., secondary) augmentation procedure using DBBM and NBCM. Subsequent implant placement was achieved in one of both patients. A conventional implant loading (cemented single crowns) was accomplished after a healing period of 8 to 16 weeks. All surgical procedures were accomplished by one operator.

### Outcome assessments

The following clinical measurements were recorded at 16 to 20 weeks after the cementation of the crown (refers to previous visit 9—new baseline) and scheduled for 0.5 (visit 1—V1), 1.5 (V2), 2.5 (V3), 3.5 (V4), and 4.5 (V5) (Fig. 1d) years after implant loading using a periodontal probe: (1) plaque index (PI) [8]; (2) bleeding on probing (BOP), evaluated as present if bleeding was evident within 30 s after probing, or absent, if no bleeding was noticed within 30 s after probing; (3) probing depth (PD) measured from the mucosal margin to the bottom of the probeable pocket; (4) mucosal recession (MR) measured from the crown margin to the mucosal margin; and (5) clinical attachment level (CAL) measured from crown margin to the bottom of the probeable pocket. All measurements were recorded at 6 aspects per implant: mesiovestibular (mb), midvestibular (b), distovestibular (db), mesiooral (mo), midoral (o), and distooral (do) by one previously calibrated investigator.

The study outline and the follow-up visits are summarized in Table 1.

**Table 1:** Days since last visit

Visit	***n***	Mean	Std	Med	Min	Q1	Q3	Max
1	9	208	130	154	126	132	237	533
2	9	301	96	315	55	308	365	365
3	8	445	107	428	254	403	550	560
4	7	314	112	335	177	232	366	490
5	5	579	106	598	432	540	603	724

*n* Number of subjects, *Std* Standard deviation, *Med* Median, *Min* Minimum value, *Q1* 25th percentile, *Q3* 75th percentile, *Max* Maximum value

### Postoperative care

At each follow-up visit, the patients were given an appropriate oral hygiene instruction and a professional implant/tooth cleaning was conducted.

### Statistical analysis

Mean values, standard deviations, medians, and frequency distributions were calculated for all clinical parameters assessed. The data rows were examined with the Shapiro-Wilk test for normal distribution. The unpaired *t* test was used for between group comparisons of the changes in mean values from baseline to V1–5.

## Results

Of the enrolled subjects, 5/9 (55.6%) completed the study, while 4/9 (44.4%) patients were lost to follow-up. A total of 42 follow-up visits were performed, 39 of those were scheduled visits and 3 were unscheduled visits. The mean number of days that passed between the visits is summarized in Table 1.

An implant survival rate of 100% could be confirmed for all patients up to V3. At V4, 77.7% (7/9) of the subjects still revealed functional implants, while no information was available for 22.2% (2/9) of the patients due to a lost to follow-up. At study completion (V5), implant survival amounted to 55.6% (5/9) of the subjects, since no information could be collected for 44.4% (4/9) of subjects due to a lost to follow-up.

### Clinical measurements

Mean and median PI, BOP, PD, and MR values measured at baseline as well as V1–5 are summarized in Table 2.

**Table 2:** Descriptive statistics of PI, BOP, PD, and MR values assessed at baseline and visits 1-5

		Baseline (***n*** = 9)	Visit 1 (***n*** = 9)	Visit 2 (***n*** = 9)	Visit 3 (***n*** = 8)	Visit 4 (***n*** = 7)	Visit 5 (***n*** = 5)
PI	mean (SD)	0.41 (0.34)	0.46 (0.39)	0.67 (0.72)	0.25 (0.40)	0.71 (0.73)	0.50 (0.58)
	median	0.33	0.50	0.33	0.00	0.33	0.50
	*P* value		0.636	0.235	0.514	0.283	0.783
BOP	mean (SD)	18.56 (37.73)	13.00 (21.7)	27.78 (32.23)	18.88 (33.48)	33.43 (33.25)	4.25 (8.5)
	median	0	0.0	17.0	8.5	17.0	0.0
	*P* value		0.399	0.280	0.794	0.029	0.391
PD	mean (SD)	2.31 (0.49)	2.48 (0.48)	3.09 (1.13)	3.62 (1.19)	3.20 (1.22)	2.96 (0.52)
	median	2.33	2.33	3.00	3.59	2.92	2.84
	*P* value		0.198	0.044	0.009	0.083	0.077
MR	mean (SD)	0.41 (0.91)	0.43 (0.87)	0.44 (0.64)	0.57 (1.52)	0.49 (1.18)	0.25 (0.50)
	median	0.0	0.0	0.0	0.0	0.0	0.0
	*P* value		0.809	0.881	0.649	0.176	0.391

Mean PI scores remained low throughout the follow-up observation period with changes to baseline amounting to 0.05 ± 0.33 at V1, 0.26 ± 0.60 at V2, − 0.08 ± 0.34 at V3, 0.26 ± 0.58 at V4, and − 0.04 ± 0.28 at V5, respectively.

Mean BOP scores changed by − 5.56 ± 18.71% at V1, 9.22 ± 23.87% at V2, − 2.00 ± 20.86% at V3, 19.14 ± 17.75% at V4, and 4.25 ± 8.50% at V5, respectively. The changes from baseline to V4 reached statistical significance (*P* = 0.029).

The changes in mean PD scores amounted to 0.17 ± 0.35 mm at V1, 0.78 ± 0.98 mm at V2, 1.33 ± 1.05 mm at V3, 0.99 ± 1.26 mm at V4, and 0.71 ± 0.54 mm at V5, respectively. The changes from baseline to V2 and V3 reached statistical significance (*P* = 0.044, *P* = 0.009).

Mean MR scores increased by 0.02 ± 0.21 mm at V1, 0.04 ± 0.71 mm at V2, 0.11 ± 0.68 mm at V3, 0.11 ± 0.18 mm at V4, and 0.25 ± 0.50 mm at V5, respectively (Table 2).

## Discussion

This study aimed at assessing the long-term clinical outcomes following lateral alveolar ridge augmentation using CXBB and staged implant placement over a period of 4.5 years.

Basically, it was noted that the surgical procedure was commonly associated with stable clinical outcomes over the entire observation period. Significant changes to baseline were merely noted for mean BOP scores at V4 (19.14 ± 17.75%, *P* = 0.029) and mean PD scores at V2 (0.78 ± 0.98 mm, *P* = 0.044) and V3 (1.33 ± 1.05 mm, *P* = 0.009), respectively.

When interpreting these results, it must be noted that the losses to follow-up at V4 and particularly V5 may limit the overall evaluation of the implant success and survival rates, which must be regarded as a major weakness of the present analysis.

Nevertheless, the clinical outcomes are within the range of previous studies also reporting on the short-/mid-term (1–3 years) and long-term (> 3 years) effects of various lateral ridge augmentation protocols on clinical implant success rates [9]. In particular, the data synthesis of the latter systematic review has pointed to minimal and non-significant BOP changes scores over time (ranging from 1 to 10 years of follow-up) [*n* = 10 publications; WMD = − 10.02%; 95% CI (− 22.23; 2.21); *P* = 0.108]. Changes in BOP were also noted when various treatment modalities (i.e., simultaneous or staged) and grafting procedures (i.e., various types of barrier membranes, growth factors, and chin blocks with or without resorbable membranes) were compared (*n* = 6; WMD = − 3.36; 95% CI [− 12.49; 5.77]; *P* = 0.471). Similar findings were also noted for changes in PD (*n* = 6; WMD = − 0.051; 95% CI 0.0; 0.0]; *P* = 0.726) [9].

When evaluating the results of the present study, it is important to emphasize that the mean ridge width at baseline was 4.38 ± 0.92 mm, which increased to 8.25 ± 2.07 mm at 24 weeks, thus resulting in a mean gain in horizontal ridge width of 3.88 ± 1.75 mm [6]. Soft tissue dehiscences were noted in 7 out of 10 patients, with an exposure of CXBB being noted in 4 patients, thus necessitating a repeated reshaping of the block during unscheduled follow-up visits until a free granulation of the dehisced area was achieved. The histological analysis of core biopsies taken during implant placement has pointed to a homogeneous osseous organization of CXBB [6]. These findings were basically in line with those data noted in a previous study also employing CXBB for lateral ridge augmentation [7]. In particular, the mean crestal width changed from 2.83 ± 0.57 mm at baseline to 6.90 ± 1.22 mm at 26 weeks, thus resulting in a mean gain in ridge width of 4.12 ± 1.32 mm. The surgical procedure, however, was also associated with a high incidence of soft tissue dehiscences (i.e., primary and secondary) (35.7%) and even early implant losses (30.8%) [7]. The histomorphometrical analysis of core biopsies pointed out that about 26.90 ± 12.21% of the evaluated surface area was occupied by mineralized vital bone, while 21.37 ± 7.36% was residual CXBB and 47.13 ± 19.15% non-mineralized tissue [10]. Higher complication rates following application of CXBB for staged lateral grafting prior to implant placement were reported in a recently published case series evaluating 5 patients [11]. In particular, a graft failure (60%) was noted in 6 out of 10 defect sites. The reported implant failure rate amounted to 33%. A successful therapy could just be noted for one out of 5 patients over an observation period of 56 months [11]. Unfortunately, the latter study did not consider the assessment of clinical outcome measures to evaluate implant success after the prosthetic restoration.

The complications associated with the usage of CXBB as reported by Schwarz et al. [6] and Ortiz-Vigon et al. [7] are within the range of the reported high incidence of wound dehiscences and graft exposures following staged lateral bone augmentation employing a variety of different materials [2]. Nevertheless, when considering the results presented by Angermaier et al. [11], its application appears to be technique sensitive.

In conclusion and within its limitations, the present clinical study revealed that CXBB was associated with high clinical implant success and survival rates on the long-term.

## Data Availability

The availability of raw data used and/or analyzed during the current study is limited/restricted by general data protection regulations.
